# Titration of DNA/Carbon Nanotube Complexes with Double-Chained Oppositely Charged Surfactants

**DOI:** 10.3390/nano5020722

**Published:** 2015-04-30

**Authors:** Franco Tardani, Camillo La Mesa

**Affiliations:** Department of Chemistry, La Sapienza University, P.le A. Moro 5, 00185 Rome, Italy; E-Mail: franco.tardani@uniroma1.it

**Keywords:** single- and double strand DNA, ss-DNA and ds-DNA, multi-walled carbon nanotubes (MWCNTs), 1/1 ss-DNA/MWCNT complexes, double-chained cationic surfactants, phase diagram

## Abstract

1/1 dispersions of ss-DNA/CNT complexes in mass ratios were investigated in a mixture with didodecyldimethylammonium bromide, DDAB. Depending on the amounts of the surface-active agent and of the complexes, solutions, precipitates, or re-dissolution occur. DDAB titrates the phosphate groups on the outer surface of the complex and controls the phase sequence in these systems. The combination of different experimental methods determined the phases that occur therein. The results are based on optical absorbance, Dynamic Light Scattering, ionic conductivity, ζ-potential, optical microscopy and AFM. From the above findings a (pseudo)-binary phase diagram is attained. The system has strong similarities with polymer-surfactant mixtures. In fact, its properties conform to cases in which interactions between rigid rod-like polyelectrolytes and oppositely charged species take place. The peculiarities of double-chained DDAB in the process imply significant differences with respect to the behavior of single chain surfactants. In fact, DDAB associates into vesicular entities, when the homologous single chain species forms small micellar aggregates.

## 1. Introduction

Colloids exhibit different organization modes, depending on whether they belong to the intrinsic or the association category [1,2,3]. In real applications the above classification loses relevance. For instance, mixing intrinsic and association colloids gives entities that do not univocally fit in any of the above definitions. Hybrid organic/inorganic colloids from sea shells [4] are examples of the above statements. Efforts were made to build up hybrid colloids by mixing those from intrinsic and association categories, in such a way that their properties self-support and self-complement [5]. Well focused experiments allow getting hybrid materials in which inorganic particles anchor bio-macromolecules. Protein-functionalized silica particles are an example [6,7].

Hybrid materials for biomedical purposes often contain inorganic silica particles [6], fullerenes [8], and carbon nanotubes [9,10,11,12]. The reasons for considering the latter species find justification in their outstanding thermodynamic stability and in mechanical and electronic properties. In addition, carbon nanotubes are easily functionalized. Biopolymer anchoring thereon is either covalent, or not [13,14,15]. We report on systems made of carbon nanotubes, CNTs, onto which single-strand DNA, ss-DNA, is anchored by non-covalent functionalization. The resulting ss-DNA/CNT complexes are stable and behave as a single entity. Their behavior is very similar to rigid rod-like polyelectrolytes. They form complexes, and monolayers, in proper conditions [16].

We mixed ss-DNA/CNT (1/1) complexes with didodecyldimethylammonium bromide, DDAB. Titration of the mentioned species with a double-chained surfactant effectively gives rise to rich polymorphic behavior. The phase sequence depends on the relative amounts of the complex and the surfactant. The observed interaction modes are similar to those of polymer-surfactant systems [17,18,19,20]. A significant novelty with respect to systems reported so far is because DDAB has two hydrophobic chains and forms vesicles, or lamellae [21,22], in place of micelles. This fact has consequences on the phase behavior and re-dissolution mechanisms.

We report on results obtained by different experimental methods, in order to clarify the intricacies inherent to the system and get information on the forces taking place therein. The forthcoming parts of the manuscript deal with: (i) preparation procedures; (ii) investigation methods; and (iii) discussion. We rely on models formerly developed for polymer-surfactant systems to analyze the results. We also report on the fundamental aspects of such mixtures, and make evident the similarities and differences met between the present and canonical polymer-surfactant systems. Similarities arise because interactions, precipitation and re-dissolution of the precipitates are analogous with the behavior met in polyelectrolyte-surfactant mixtures [23].

## 2. Results and Discussion

### 2.1. Optical Absorbance

**Figure 1:**
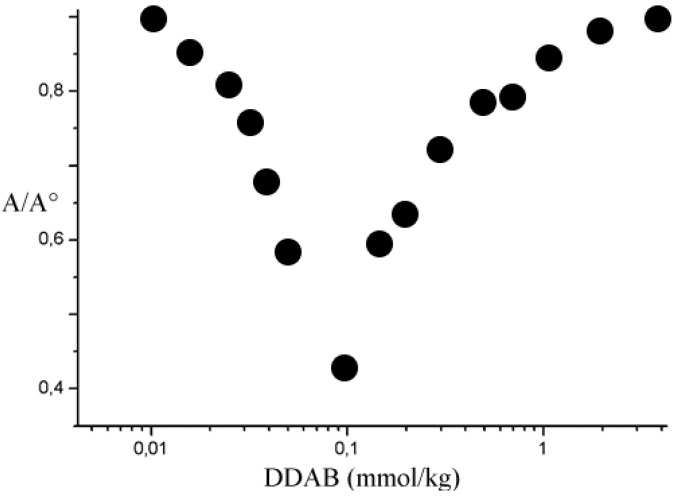
Normalized optical absorbance at 660 nm of a dispersion containing 0.01 wt.% of 1/1 ss-DNA/CNT complex upon titration with DDAB, at 25.0 °C. The data accuracy is within the symbols size.

Titration implies charge neutralization and precipitation. Upon precipitation the turbidity decreases and reaches a minimum when the process goes to completion (Figure 1). Similar curves similar are observed at all concentrations. The minimum in the plots, however, becomes more shallow the lower the 1/1 ss-DNA/CNT concentration.

The precipitates are sticky when touched by hand, presumably because extended hydrophobic moieties cover them. The addition of DDAB results in the re-dissolution of the precipitates. We do not yet know whether the process is micelle- or vesicle-assisted, and support from dynamic light scattering, DLS, is required.

### 2.2. DLS

The average aggregates size changes in proportion to DDAB content. The maximum size and dispersity occurs at the neutralization threshold, where sizes as high as 400 nm occur. The poly-dispersity reaches a maximum at charge neutralization and levels off thereafter. Neutralized complexes are larger than vesicles and complexes (200 nm each). Information from DLS goes parallel to optical absorbance. Data combination supports particle growth upon neutralization.

### 2.3. ζ-Potential

**Figure 2:**
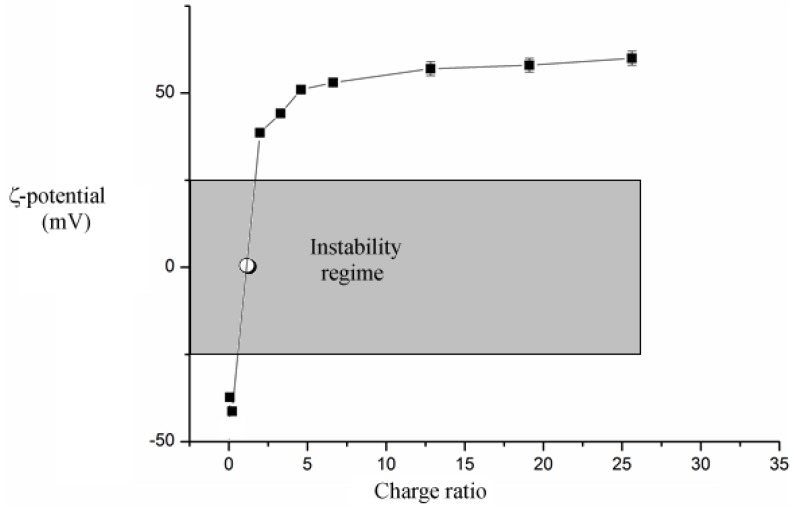
Dependence of ζ-potential values, at 25.0 °C, on the nominal charge ratio between complexes and DDAB. The white symbol indicates charge neutralization, the grey area is the instability regime. The accuracy on the individual points is within the symbols’ size; the line is for visual purposes.

**Figure 3:**
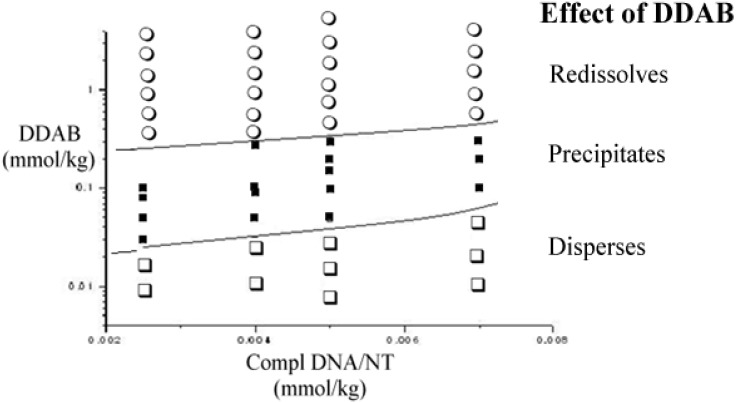
Partial phase map, inferred by optical absorbance, Dynamic Light Scattering (DLS) and ζ-potentials, at 25.0 °C. The location of the phase borders is tentative. Data are reported as the concentration of 1/1 ss-DNA/CNT complex *vs.* that of DDAB. Errors are lower than the symbols size.

Changes in surface charge density upon neutralization are significant. Before the addition of DDAB the surface charge density of complexes is negative. Titration leads to a significant decrease in the modulus of ζ. The latter approaches zero at charge neutralization, Figure 2, and becomes positive and constant above that point. The value at the charge inversion threshold is a “true” phase separation. Precipitation may occur at ζ-potential values slightly different from zero, as indicated in Figure 3. In fact, values lower than 25 mV (in modulus) do not ensure kinetic stability to the dispersions.

### 2.4. Ionic Conductivity

Two changes in slope should occur in conductivity *vs.* DDAB plots; one at a surfactant content close to the critical aggregation, CAC, the second at the critical micellar concentration, CMC*. In the present experimental conditions the former point is missing. The reason is due to the fact that the CAC is in the range 10^−5^–10^−4^ mol kg^-1^ [21], and is lower than the CMC. The increase in conductivity due to DDA^+^, the cation, and Br^-^ ions is extremely moderate in such concentration regimes. Therefore, the questionable determination of CAC is due to the small increments in conductivity in a medium containing substantial amounts of ions.

**Figure 4:**
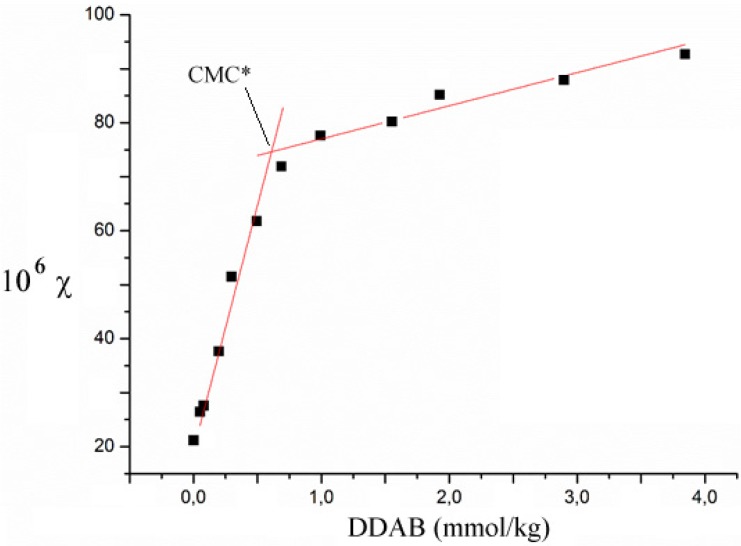
Conductivity, χ (μS cm^−1^), *vs.* concentration plot for a 1/1 ss-DNA/CNT system, at 0.010 wt.%, to which progressive amounts of DDAB, in mmol kg^−1^, are added. Data are taken at 25.0 °C.

Therefore, the behavior reported in Figure 4 occurs. A critical threshold, termed CMC*, is observed, but not the CAC, for the reasons given above. The intercepts and slopes of conductivity depend on ss-DNA/CNT content. Conductivity increments below the CMC* are ascribed to the release of sodium ions from ss-DNA/CNT complexes. The intersection of conductivity curves lies in the range 0.5–0.7 mmol kg^−1^. The measured CMC* can be over one order of magnitude higher than the CMC [19,20]. Please note that the critical values inferred by conductivity are close to the re-dissolution limits inferred by turbidity and DLS.

### 2.5. Optical Microscopy and Atomic Force Microscopy (AFM)

**Figure 5:**
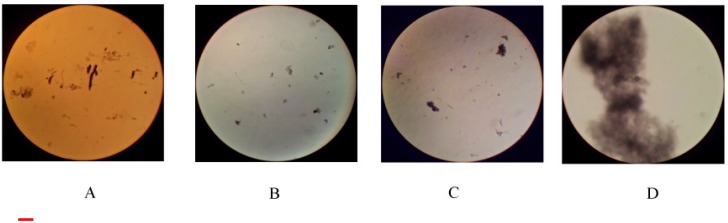
Optical micrographs of samples containing 0.0050 wt.% of 1/1 ss-DNA/CNT complexes and variable amounts of DDAB, at 25.0 °C. Magnification is 40× in pictures (**A**–**C**); 400× in (**D**). Figure (**A**) refers to 0.15; (**B)** to 0.29; (**C**) and (**D**) to 0.098 mmol·kg^−1^, respectively. The red bar in the lower left hand side of the figure is 1.0 mm large.

Such investigations determined the coarse and fine morphology of the entities occurring herein. The interactions between the components gives rise to peculiar micro- and nano-scopic features, interrelated to each other. It is possible to observe indented particles or large and densely packed ones. The latter are substantially unstable and tend to precipitate. As an example, we report pictures relative to 0.005 wt.% in ss-DNA/CNT, in 1/1 mass ratio, and progressive amounts of DDAB, Figure 5. The size and appearance of precipitates depend on the mole ratio between reactants; in addition, long equilibration times do not affect their maturing. However, re-dissolution is cumbersome when samples are concentrated. The role of DDAB on association kinetics is not clear. The first stage of the process, presumably due to surfactant adsorption/binding, is fast. Nucleation and precipitation take place thereafter.

**Figure 6:**
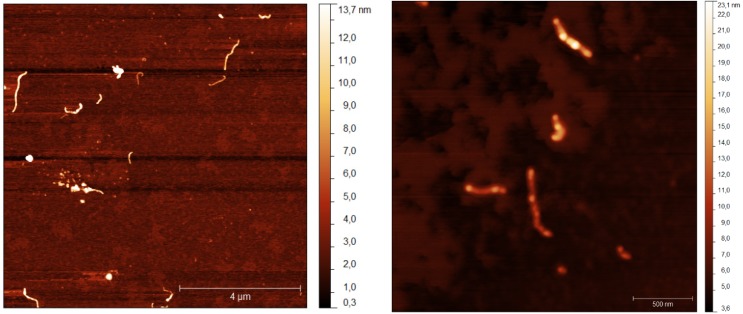
Atomic Force micrographs of samples containing 0.0050 wt.% of 1/1 ss-DNA/CNT complexes with no, left, and 0.4 mmol kg^−1^ DDAB. The size of images is inferred by comparison with bars in the right bottom side of both figures.

Atomic Force Microscopy (AFM) clarified that bundles 15 nm large are present, Figure 6. No large aggregates occur in such conditions. Uncharged colloid entities precipitate and get compact thereafter. To date, the effective phase separation pathways are not fully understood, since an explanation of all forces in play is missing.

## 3. Discussion

To shed light on the above items and to make easy a full understanding, we first a brief description on polymer-surfactant systems. This is because of the strong similarity that ss-DNA/CNT complexes have with polyelectrolyte-surfactant systems, PSS.

### Polymer-Surfactant Systems (PSS)

**Figure 7:**
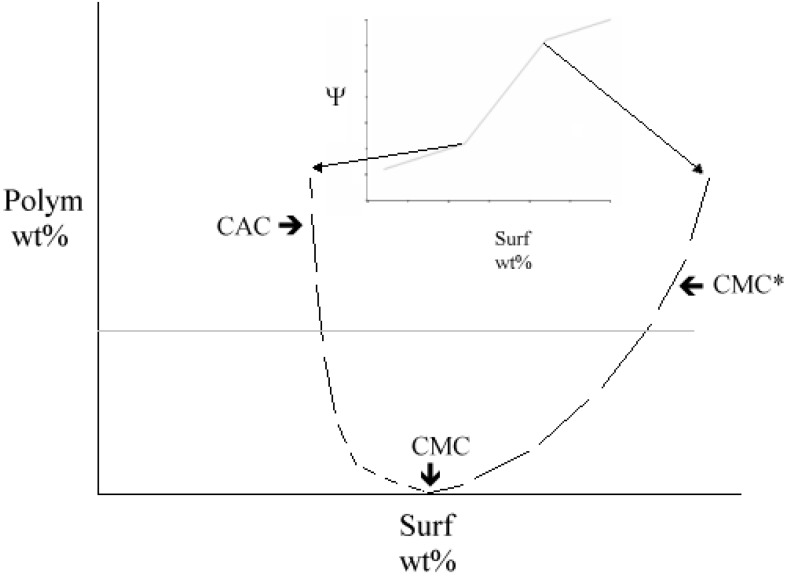
The behavior inherent to polymer-surfactant systems. Critical aggregation, CAC, and critical micellar concentration, CMC*, curves meet at the CMC, indicated in the bottom of figure. In the inset the behavior of a physico-chemical property, Ψ, *vs.* surfactant content, at fixed polymer wt.% is reported. Two changes in slope occur at the corresponding critical points.

In PSS a polymer interacts with a surfactant. The solution properties are controlled by the relative amounts of the components; as a rule, a rich phase behavior can be met [21,22,23]. In the presence of double chained surfactants no such information is available. A rich polymorphic behavior is supposed to occur, since association of the adduct is concomitant to the formation of many different regions in the phase map [24,25,26]. In the presence of polymers the associative behavior of surfactants splits in three regions, of which the central one may be a solution, a gel, or a precipitate. At low concentrations, the polymer and the surfactant do not interact and solutions occur. In intermediate regimes, the surfactant adsorbs onto the polymer and binds therein. Once the polymer binding sites are saturated, free micelles do form, Figure 7. The latter entities coexist with polymer-surfactant complexes and, presumably, re-dissolve them.

The separation thresholds between the different states are indicated as critical aggregation, CAC, and critical micellar concentration, CMC*, respectively [19]. The surfactant partition depends on the number of binding sites available on the macromolecule, and on its concentration. The number of surfactant molecules on each binding site depends on the polymer and on the surfactant. As a rule, the number of molecules in each binding site is lower than that pertinent to free micelles. The interactions are electrostatic, hydrophobic, and/or combinations thereof.

The width of the interaction region depends on the polymer wt.%, nature, functional groups, and by the surfactant affinity toward it [27]. The overall surfactant content, *X*_tot_, is the sum of molecularly dispersed, *X*_1_, free micelles, *X_m_*, and polymer-bound entities, *X_p_*_,*b*_, respectively. Micelles contain, on the average, *n* units and *X_m_* = *n*(*K_m_X*_1_)*^n^*; the third term is *X_p_*_,*b*_ = *mLX*_pol_[(*K_p_*_,*b*_*X*_1_)*^m^*/1 + (*K_p_*_,*b*_X_1_)*^m^*]. This equation presents m as the number of surfactant molecules adsorbing on a binding site and *L* of which are present in a given polymer [28]. The binding of a single aggregate per site holds. In addition, *K_m_* and *K_p_*_,*b*_ indicate the equilibrium constant for the formation of free and polymer-bound aggregates, respectively. Furthermore, *n* ≠ *m*, since micelle sizes are different in the two cases.

*K_p_*_,*b*_, *K_m_*, *n* and *m* values in the equations determine whether complex formation occurs, or prevails with respect to micelle onset. When *K_m_*< *K_p_*_,*b*_ and *n* ≈ *m*, complex formation and aggregation on the polymer binding sites takes place first; thus, free aggregates prevail. We do not consider the possibility that *m* << *n*. That statement would imply aggregates onset before saturation; no such evidence was observed. For a given amount of polymer, *X*_pol_, the mass balance gives

(1) $$X_{tot}=X_{1}+\text{n}{\left(K_{m}{\text{X}}_{1}\right)}^{n}+{\text{mLX}}_{pol}\left[{\left({\text{K}}_{p,b}{\text{X}}_{1}\right)}^{m}/1+{\left({\text{K}}_{p,b}{\text{X}}_{1}\right)}^{m}\right]$$

Equation (1) requires some approximations. One assumes *K_p_*_,*b*_ > 1/*X*_1_ and imposes linearity in *X*_pol_. A second alternative arises from the derivation of an experimental quantity with respect to *X*_pol_. *X*_1_ depends on the polymer content in an undefined way. At a given *X*_pol_, the (CMC*/CAC) ratio depends on the surfactant affinity toward the polymer binding sites and corresponds to a partition coefficient, *K*_Part_. The highest is *K*_Part_ due to the wider the interaction region and, therefore, number of binding sites, Figure 7 [29,30,31]. Free micelles do form when almost all binding sites are saturated. The process depends on the complex and on the conformational states allowed to it. On such grounds, 1/1 ss-DNA/CNT complexes are similar to double strand DNA, ds-DNA [32,33]. Therefore, similarities between complexes and ds-DNA are a realistic working hypothesis.

Deriving Equation (1) with respect to *X*_pol_ gives an overview of the process. That procedure, perhaps, does not separate electrostatic and hydrophobic contributions to binding. A simple way to overcome such drawbacks relies on the comparison with a species having features in common with DDAB. For these reasons, the behavior of dodecyltrimethylammonium bromide, DTAB [32], is compared to that of DDAB. They have the same nominal charge and main alkyl chain length, but hydrophobic contributions to binding are quite different in the two systems. DTAB, in fact, has a very moderate capacity to redissolve the precipitates, even though it is in substantial concentrations.

We refer to turbidity data, which indicate that the process is cooperative and goes to completion at charge neutralization. The number of available binding sites is proportional to (∂A/∂DDAB). According to data, a small discontinuity in A *vs.* DDAB plots occurs in the 1.0 × 10^−5^ mol kg^−1^ range; presumably, this is the CAC. DDA^+^ adsorbs onto complexes and forms large hydrophobic domains. The observed behavior resembles those indicated in “Small Systems Thermodynamics” [33], where each complex is a small system. This hypothesis has been widely used in polymer-surfactant systems [34].

Binding models are based on statistical thermodynamics [35], or chemical approaches [36,37]. They suggest that binding occurs onto many equivalent sites. Presumably, this results in cooperative or anti-cooperative interaction modes. Each possibility depends on whether DDA^+^ is clustering on a binding site, or steric repulsion between two adjacent DDA^+^ ions occurs. The former hypothesis holds when strong hydrophobic interactions take place. In words, the bulky DDA^+^ favors the binding of a second ion. Thus, clustering around a neutralized site is energetically favored. Their formation depends on the cooperativity parameter, *u*, and binding degree, β, where the latter is in the range 0.2–0.8. For a given set of values we calculated the average number of DDA^+^ ions in a cluster, *m*, according to [38,39].

(2) $$\text{m}=2\beta \cdot \frac{\left(u-1\right)}{\left\{{\left[4\beta \cdot \left(1-\beta \right)\cdot \left(u-1\right)+1\right]}^{\frac{1}{2}}-1\right\}}$$

We also imposed *u* to be

(3) $$u=exp^{\frac{\left[2E_{a,b}-E_{a,a}-E_{b.b}\right]}{kT}}$$

In Equation (3), *E_a_*_,*a*_, *E_a_*_,*b*_ and *E_b_*_,*b*_ are the interaction energies of free, *E_a_*_,*a*_, and fully, *E_b_*_,*b*_, or partly, polymer-bound states, respectively. The difference amounts to *E*_tot_. In the calculations, we imposed that *E*_tot_/*kT* is 5, 10, or 20 units. These values are not always in the above range. In the present conditions, *m* increases in proportion to β and *u*, whereas binding is continuous and not superiorly limited. It is conceivable that bound surfactant ions favor the clustering of amphiphilic molecules into aggregates.

**Figure 8:**
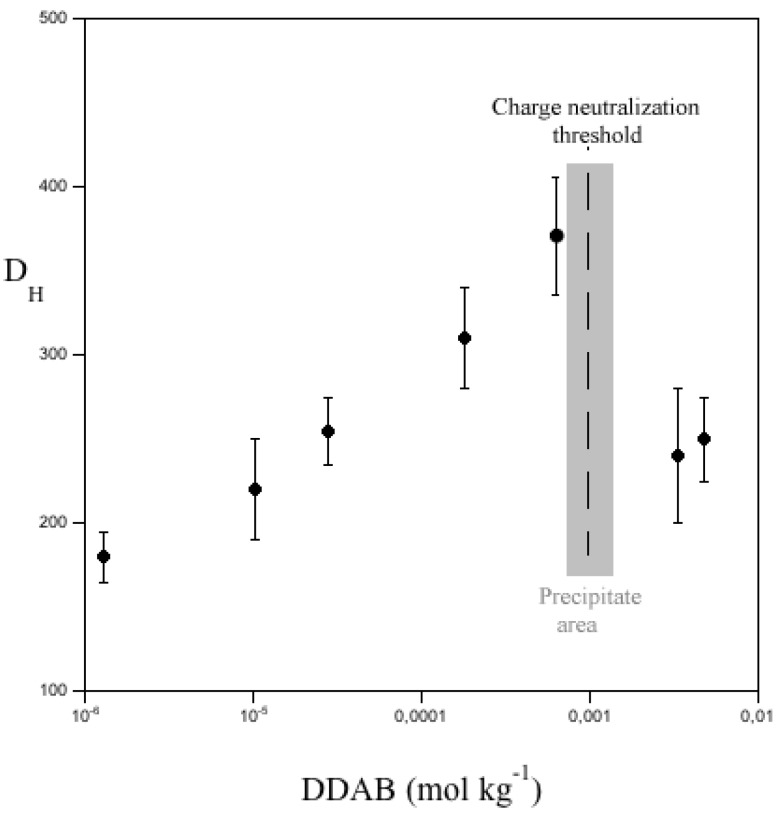
Average size of ss-DNA/CNT complexes, *D_H_*, in nm, versus the concentration of added DDAB, in mmol kg^−1^, at 25.0 °C.

The size of polymer-bound aggregates is always lower than that of free micelles [26,40]. In fact, the Gibbs energy terms for free and bound states are different; in particular, curvature and electrostatic terms are not the same in the two cases. In presence of DTAB (the single chain homologue of DDAB), the redissolution of precipitates is moderate, if any. It slightly depends on the amount of surfactant in micellar form [34]. Therefore, the behavior of a single chain species is significant less effective than double-chained ones. We could not evaluate the size of the adducts by fluorescence, given the intense dark color of the dispersions, the presence of ss-DNA/CNT complexes, and the large size of DDAB-based aggregates. According to DLS, however, the size of DDAB-titrated complexes reaches a maximum at charge neutralization and levels off thereafter, Figure 8. *D_H_* values therein result from the contributions due to all scattering entities: bare complexes, adducts, and micelles, or, presumably, vesicles. The maximum *D_H_* value corresponds to the inflection points in conductivity and optical absorbance curves. In addition, optical microscopy and AFM indicate that complexes grow in size upon titration with DDAB; more surfactant re-dissolves the sticky aggregates. Meso- and macroscopic sizes are the result of a complex balance between hydrophobic and electrostatic contributions. The latter increase in direct proportion to DDAB, up to precipitation; in the same time, charge neutralization modulates the resulting interactions. Saturation and charge neutralization are not solely responsible for the system stability. Surface effects, osmosis, depletion [41], and other terms surely play an important role in re-dissolution.

## 4. Experimental Section

### 4.1. Chemicals

Didodecyldimethylammonium bromide, DDAB, was a 98% nominal purity product, Sigma Aldrich, Milan, Italy. It was dried before use. Its phase behavior and solution properties allowed researchers to detect its purity through comparison with literature data [21,42]. Calf thymus double strand DNA, ds-DNA, was from Sigma Aldrich and transformed in the single strand form, see below for details. Water was doubly distilled over alkaline KMnO_4_ and degassed before use, to avoid CO_2_ uptake. Its ionic conductivity is lower than 1.0 × 10^−7^ Ω^−1^ cm^−1^, at 25.0 °C. Multi-walled carbon nanotubes, MWCNTs, SouthWest Nano Technologies, are also from Sigma Aldrich. Their nominal purity is 98% on carbon basis. According to the purveyor, they have an average outer diameter of 10 ± 1 nm and lengths in the 3–6 μm range. The results were confirmed by TEM [43].

### 4.2. Materials preparation

Double strand DNA solutions were heated at 90 °C, kept there for 45 min, and quenched at 20.0 °C through immersion in a thermostatic water bath [33]. In this way, ds-DNA is transformed in its single-strand form, ss-DNA. MWCNTs are added with ss-DNA, to get a 1/1 mass ratio between solutes. A Vibracell VC300 unit, working at 20 kHz, sonicated the dispersions for 2 h. The amount of nanotubes in the dispersing medium is 0.01 wt.%. Diluted samples were prepared by adding water. The dispersions homogeneity is checked by light microscopy; no bundles or large particles were observed.

## 5. Methods

### 5.1. Optical Microscopy

A Ceti Laborlux unit checked the state of the dispersions, in normal and/or polarized light, at 25.0 °C. Samples were located on cleaned glass slides; thickness was controlled by inserting Teflon spacers between slides and cover-slides. Shear was applied parallel to the major slide axis. Samples were sandwiched between slides and covered by epoxy resins, to keep composition constant. Pressing determined the textural evolution subsequent to deformations.

### 5.2. DLS

Measurements were run by a Malvern Zeta Nanosizer, at 632.8 in back scattering mode (at 173°), and 25.0 °C. A correlator analyzed the light intensity fluctuations, *I*(*q*,*t*), due to Brownian motions. Intensity was evaluated by the autocorrelation function, *G*_2_(*q*,*t*), through CONTIN [44]. *G*_2_(*q*,*t*), is obtained according to

(4) $$G_{2}\left(q,t\right)=\frac{\langle \left[I\left(q,t\right)\right]\left[I\left(q,t+\tau \right)\right]\rangle \;\;\;\;}{{\langle \left[I\left(q\right)\right]\rangle }^{2}\;}$$

where *q* is the scattering vector, τ the delay time, and braces indicate the time average. Equation (5) is transformed in the field autocorrelation function, *g*_1_(*q*,*t*), as

(5) $$G_{2}\left(q,t\right)=\;A_{1}+B{\left\lceil q_{1}\left(q,t\right)\right\rceil }^{2}$$

*A* is the baseline and *B* depends on the aggregates. *g*_1_(*q*,*t*) is expanded in cumulants, to give

(6) $$ln\left|q_{1}\left(\tau \right)\right|=-\Gamma_{1}\tau +\left(\frac{\Gamma_{2}}{2}\right)\tau^{2}$$

Γ_1_, the first cumulant, provides information on the particle(s) self-diffusion, *D*, and on their average hydrodynamic radii. Γ_2_, conversely, is related to the poly-dispersity index, PdI.

### 5.3. ζ-Potential

Measurements were run by a Laser-Doppler facility available in DLS unit. The apparatus operates with cells equipped with gold electrodes, at 25.0 °C. ζ-potential, ζ, is inferred by electrophoretic mobility, μ, according to [45,46]:

(7) $$\zeta =\mu \left(\frac{4\pi \eta }{\varepsilon \prime }\right)$$

where η is the solvent viscosity and ε’ its permittivity. In all cases considered here, Smoluckowski’s approximation holds; thus, the electrical double layer thickness around particles, δ, is much lower than their hydrodynamic radius [47].

### 5.4. AFM

It was run with a Bruker AXS unit. Images were acquired in air, at room temperature, in tapping mode. A high-resolution RTESP (Rotated Tapping Etched Silicon) probe from VEECO Probes USA was used. A tip with a radius of curvature, *R*, < 8 nm insists on a rectangular 125 μm long cantilever, with resonant frequency of 300 kHz and spring constant equal to 40 N m^−1^. The samples are deposited on cleaved mica, incubated, rinsed with Milli-Q water, flushed with nitrogen and analyzed within 30 min. Images were determined by Gwiddion facilities.

### 5.5. Optical Absorbance

Measurements were run by a Jenway 6400 spectrophotometer. Samples were centrifuged, and the supernatant examined by turbidity. Centrifugation induces the precipitation of complexes and/or bundles and promotes the aggregation of unstable entities. Absorbance was measured at 660 nm, to avoid interference with DNA. Each run is in triplicate. Values were normalized for the absorbance of the mother dispersion. The A/A° ratio is proportional to the amount of complexes remaining in the medium, Figure 1.

### 5.6. Ionic Conductivity

A Wayne-Kerr Unit, model 6425, equipped with a 1.0 cm^3^ conductivity cell, measured ionic conductance, κ. The cell is thermostated at 25.000 ± 0.002 °C by a circulating oil. Stirring of the dispersions reduces concentration gradients. Known aliquots of DDAB were added with a weight burette. An F25 thermometer, Automatic System Laboratories, ensured the constancy of temperature.

## 6. Conclusions

Single strand DNA/CNT complexes were prepared and reacted with DDAB. Upon titration, the complexes phase separate, and re-dissolve when the surfactant content is higher than that required for neutralization. The phase behavior of the present system strongly resembles that of polyelectrolyte/surfactant systems [48], in cases where precipitation (or gelling) and re-dissolution take place [49]. Surfactant binding is responsible for both precipitation and re-dissolution, depending on the relative amounts of the two species. In consequence of these mutual interactions, the system forms sticky and non-soluble complexes, which tend to associate in large particles or films [50]. Apparently, the processes do not induce conformational changes in ss-DNA/CNT complexes.

CMC* values were determined by different experimental methods, which univocally indicate re-dissolution [48]. Given the extremely low critical concentration of DDAB, it was not possible to detect the CAC from experiments. We estimated the binding efficiency and compared it with values obtained with DTAB as counter-ions [34]. Comparison among data indicates that the behavior of DDAB is much more effective than the single chain homologue, as expected from the significant differences in hydrophobic contributions. The binding energy is significant and co-operative. In fact, precipitation after charge neutralization is almost complete, with the formation of sticky flakes. In the present system, therefore, polymer-surfactant interactions dominate over micelle, or vesicle formation.

The present findings offer the opportunity to get significant information on both fundamental and applied aspects. It comes out that the role of hydrophobic interactions, subsequent to DDA^+^ anchoring onto the complexes, is noticeable. This fact has relevant consequences in a lot of practical applications. Those related to the anchoring of selected biologically relevant molecules onto DNA-based complexes are of substantial interest in bioassays.
